# Exportin 4 Interacts with Sox9 through the HMG Box and Inhibits the DNA Binding of Sox9

**DOI:** 10.1371/journal.pone.0025694

**Published:** 2011-10-03

**Authors:** Megumi Tsuchiya, Hidesato Ogawa, Taiga Suzuki, Noriyuki Sugiyama, Tokuko Haraguchi, Yasushi Hiraoka

**Affiliations:** 1 Graduate School of Frontier Biosciences, Osaka University, Suita, Japan; 2 Division of Sex Differentiation, National Institute for Basic Biology, National Institutes of Natural Sciences, Okazaki, Japan; 3 Advanced ICT Research Institute Kobe, National Institute of Information and Communications Technology, Kobe, Japan; 4 Neuroscience Research Institute, University of California at Santa Barbara, Santa Barbara, California, United States of America; 5 Kyoto Prefectural University of Medicine, Kawaramachi-dori, Kyoto, Japan; University of Saarland Medical School, Germany

## Abstract

Sox9 is a transcription factor that is required for tissue development in mammals. In general, such transcription factors require co-regulators for precise temporal and spatial control of the activation and inactivation of the numerous genes necessary for precise development during embryogenesis. Here we identify a new Sox9 co-regulator: Using affinity chromatography with immobilized Sox9 protein, we identified exportin 4 (Exp4) as an interacting protein of Sox9 in human cultured cells. Interaction between endogenous Exp4 and Sox9 proteins was confirmed in the human osteosarcoma U2OS cells by immunoprecipitation experiments using anti-Sox9 antibody. siRNA depletion of Exp4 enhanced transcription of Sox9 target genes in U2OS cells, but did not affect nuclear localization of Sox9. These results suggest that Exp4 regulates Sox9 activity in the nucleus. Furthermore we found that the HMG box of Sox9 was responsible for binding to Exp4, and the HMG box was required for suppression of Sox9-mediated transcription. This contrasts with the known Sox9 co-regulators which bind to its transcriptional activation domain. Chromatin immunoprecipitation analyses revealed that Exp4 prevents Sox9 binding to the enhancers of its target genes. These results demonstrate that Exp4 acts as a Sox9 co-regulator that directly regulates binding of Sox9 to its target genes.

## Introduction

Sox family genes encode transcription factors and belong to a superfamily of genes characterized by a homologous sequence called the high-mobility group (HMG) box. The HMG box is a DNA-binding domain that is highly conserved between species. A family of 20 Sox genes is present in humans and mice. The proteins they encode are divided into 10 subgroups, named alphabetically from A to J, according to their HMG domain homology [1].

The Sox HMG domain is known to bind to specific DNA sites, and to thereby activate transcription of target genes [2]. In addition, the domain has a role in the subcellular localization of the Sox proteins [3]. Independent nuclear localization signals (NLS) have been identified at each end of the HMG domain, designated the N-terminal NLS and the C-terminal NLS; these NLS regions may bind different factors. [4], [5]. A nuclear export signal (NES) has also been identified within the HMG domain, in the region located between the two NLSs [6].

Sox9 is a member of group E and has a role in tissue-specific development such as chondrogenesis and male gonadal development. It has been shown that heterozygous defects in the human Sox9 gene lead to campomelic dysplasia, a skeletal malformation syndrome, which is often linked to XY sex reversal syndrome [7], [8]. In chondrogenesis, a minimal DNA element in intron 1 of the gene is responsible for chondrocyte-specific expression of type II collagen alpha 1 (Col2a1) which is abundant in the early steps of chondrocyte differentiation [9]. This element, which has strong chondrocyte-specific enhancer activity, is a direct target of Sox9 [10].

Several Sox9-interacting proteins have been identified as factors which regulate the transcriptional activity of Sox9, e.g., TRAP230 protein [11], CREB-binding protein (CBP)/p300 [12], PGC-1α [13], PIAS proteins [14] and Tip60 [15]. All these proteins interact with Sox9 through binding to a C terminal region of Sox9, the transcriptional activation (TA) domain [16], [17]. These interactions contribute to the regulation of Sox9-mediated transcription at the Sox9-specific target promoter [18], [19].

In order to understand the molecular mechanisms of Sox9-dependent transcriptional activation in more detail, we performed *in vitro* pull-down experiments to identify other major interaction partner proteins of Sox9. We identified Exp4 as a Sox9-binding protein by mass spectrometry analysis. Exp4 is a member of the importin β superfamily and is known to export eukaryotic translation initiation factor 5A (eIF-5A) and Smad3 to the cytoplasm in a Ran-dependent manner [20], [21]. Furthermore, we found that Exp4 binds to the HMG box of Sox9, unlike the previously indentified co-regulators, and suppresses transcriptional activity by inhibiting DNA binding of Sox9. Here we demonstrate that Exp4 is a modulator of Sox9-mediated transcription.

## Results

### Identification of proteins that interact specifically with Sox9

**Figure 1:**
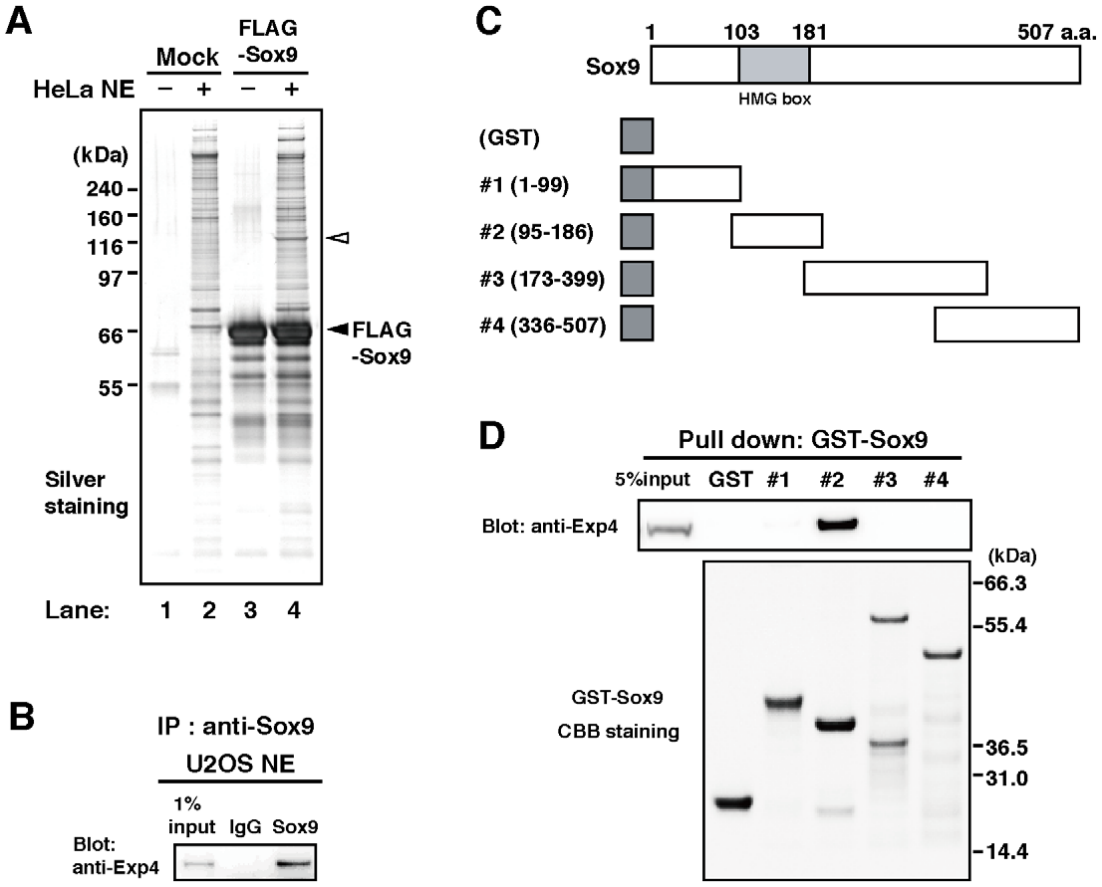
Identification of Exp4 as a major interaction partner of Sox9. (A) Silver staining of Sox9 binding proteins separated by NuPAGE. Nuclear extracts prepared from HeLa cells (HeLa NE) were incubated with (lanes 3, 4) or without FLAG-tagged Sox9 (FLAG-Sox9, lanes 1, 2). After recovery with anti-FLAG M2 antibody-conjugated agarose, the proteins were subjected to NuPAGE. The closed arrowhead indicates FLAG-Sox9, and the open arrowhead indicates the protein that was specifically recovered by FLAG-Sox9 (lane 4). (B) Nuclear extracts from U2OS cells were subjected to immunoprecipitation with anti-Sox9 antibody, and the precipitates were subjected to Western blotting analysis using anti-Exp4 antibody (right lane). Normal rabbit IgG was used as a control (middle lane). 1% of the nuclear extract was applied as a control (left lane). (C) The schematic depicts the truncated forms of Sox9 fused with GST (dark gray boxes). The numbers indicate the amino acid residues. The HMG box domain is shown as a light gray box (103–181 a.a.). (D) The upper panel shows Western blotting analysis of the protein samples co-precipitated with GST-fused truncated forms of Sox9 using an anti-Exp4 antibody. 5% of the nuclear extract was applied as a control (left lane). Numbers represent the corresponding GST-fused truncated Sox9 constructs shown in C. The lower panel shows CBB staining of NuPAGE for the GST fusion proteins used in this experiment. Numbers on the right represent the molecular weights of the marker proteins.

To identify proteins that specifically interact with Sox9, full length FLAG-Sox9 immobilized on beads was incubated with HeLa cell nuclear extract and the bound proteins collected and analyzed. NuPAGE analysis identified a protein that had specifically bound to Sox9 (see the open arrowhead in Figure 1A). The protein contained in this band was analyzed by matrix assisted laser desorption ionisation time-of-flight mass spectrometry (MALDI–TOF MS), and identified as Exp4. To further confirm the interaction between Sox9 and Exp4 in physiological conditions, we performed immunoprecipitation experiments for the proteins endogenously expressed in cultured cells using anti-Sox9 antibody. We found that Sox9 co-immunoprecipitated with Exp4 (Figure 1B). These results suggest that endogenous Sox9 binds to Exp4 in the cell.

### The HMG box domain of Sox9 is responsible for the interaction of Sox9 with Exp4

To identify the molecular domains of Sox9 that interact with Exp4, GST-tagged fragments of Sox9 were prepared (Figure 1C, D) and subjected to pull-down assays using nuclear extracts from HeLa cells (see *Materials and Methods* for details). Western blotting analysis with anti-Exp4 antibody showed that region #2 (95–186 a.a.), which carries the DNA binding HMG box domain, bound to Exp4, whereas regions #1 (1–99 a.a.), #3 (173–399 a.a.) and #4 (336–507 a.a.), and GST alone did not (Figure 1D). These results indicate that the HMG box domain of Sox9 is responsible for the interaction of Sox9 with Exp4.

### Interaction of Exp4 with other members of the Sox family

**Figure 2:**
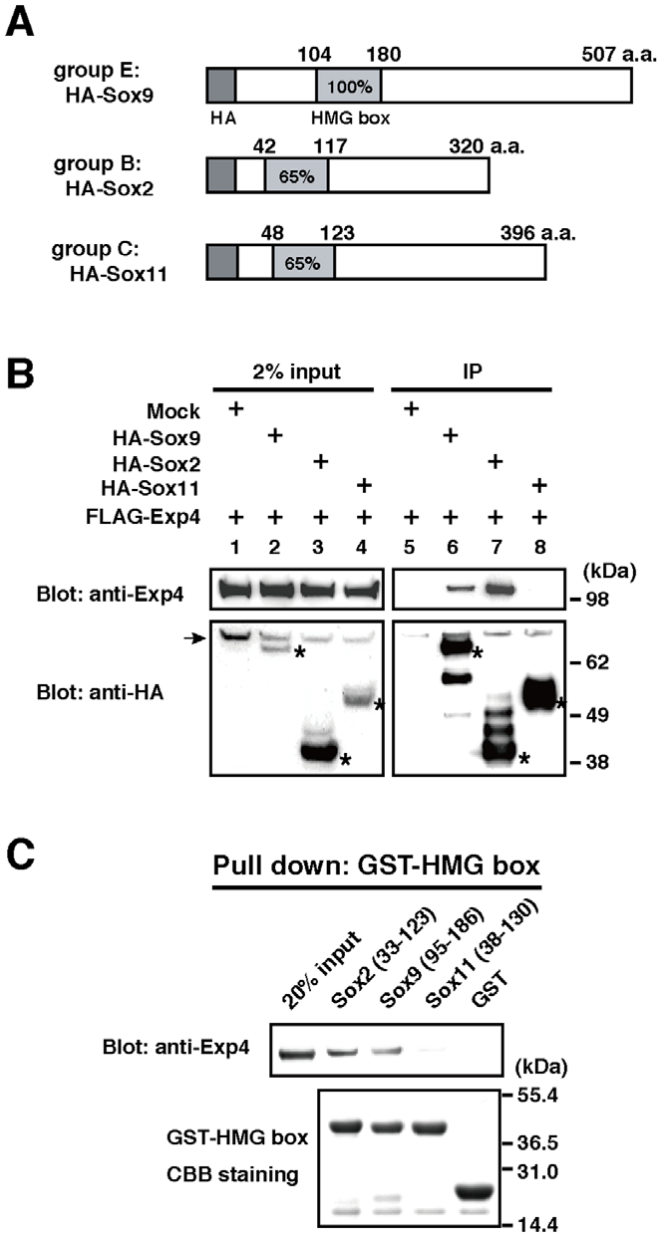
Interaction of Exp4 with Sox family members. (A) Schematic representation of HA-tagged Sox proteins used in this study. The numbers indicate the amino acid residues. The HMG box domain is shown as a light gray box. The percentage of amino acid identity with the amino acid sequence of the HMG domain of Sox9 is given. (B) The panels show HA-affinity purification of proteins from extracts of HEK293 cells which were transiently transfected with FLAG-Exp4 and HA-Sox9, HA-Sox2, or HA-Sox11. Mock refers to the empty control plasmid. Starting materials (2% input) and bound fractions (IP, immunoprecipitation) were analyzed by NuPAGE and Western blotting. HA-tagged proteins are asterisked in the lower panels. The arrow indicates nonspecific bands. (C) The GST-fused HMG box domains of each Sox protein were separated by NuPAGE and stained with CBB (lower panel). The fusion proteins were incubated with recombinant Exp4 proteins. Proteins bound to glutathione-Sepharose were analyzed by Western blotting with anti-Exp4 antibody (upper panel). 20% input represents the control.

As a recent report shows that Exp4 interacts with Sox2 and SRY, both members of the HMG box family of proteins [22], we examined whether Exp4 interacts with other subgroups of the Sox family proteins: Sox9 (from group E), Sox2 (from group B), and Sox11 (from group C). To test if they bind to Exp4 in the cell, HA-tagged Sox9, Sox2, or Sox11 (Figure 2A) was expressed together with FLAG-Exp4 in HEK293 cells and the cell lysates were subjected to immunoprecipitation experiments using anti-HA antibody (Figure 2B). Western blotting analysis of the immunoprecipitates showed that HA-Sox9 and HA-Sox2, but not HA-Sox11, co-immunoprecipitated with FLAG-Exp4 (lanes 6–8 in Figure 2B), indicating that Exp4 binds to limited set of Sox protein family members.

As our experimental data show that the HMG box domain of Sox9 is responsible for binding with Exp4 (see Figure 1D), we next examined whether differences in the ability of the Sox family proteins to bind Exp4 reflected differences in their HMG box domains. GST-tagged HMG box fragments of Sox2, Sox9 and Sox11 were prepared and subjected to GST pull-down assays with purified recombinant FLAG-Exp4 protein. The Sox2 fragment (33–123 a.a.) and the Sox9 fragment (95–186 a.a.) directly bound Exp4, whereas the Sox11 fragment (38–130 a.a.) and GST alone did not (Figure 2C). These results indicate that the HMG box domain is primarily responsible for binding of Sox family proteins to Exp4.

### Effect of Exp4 on Sox9-mediated transcription

**Figure 3:**
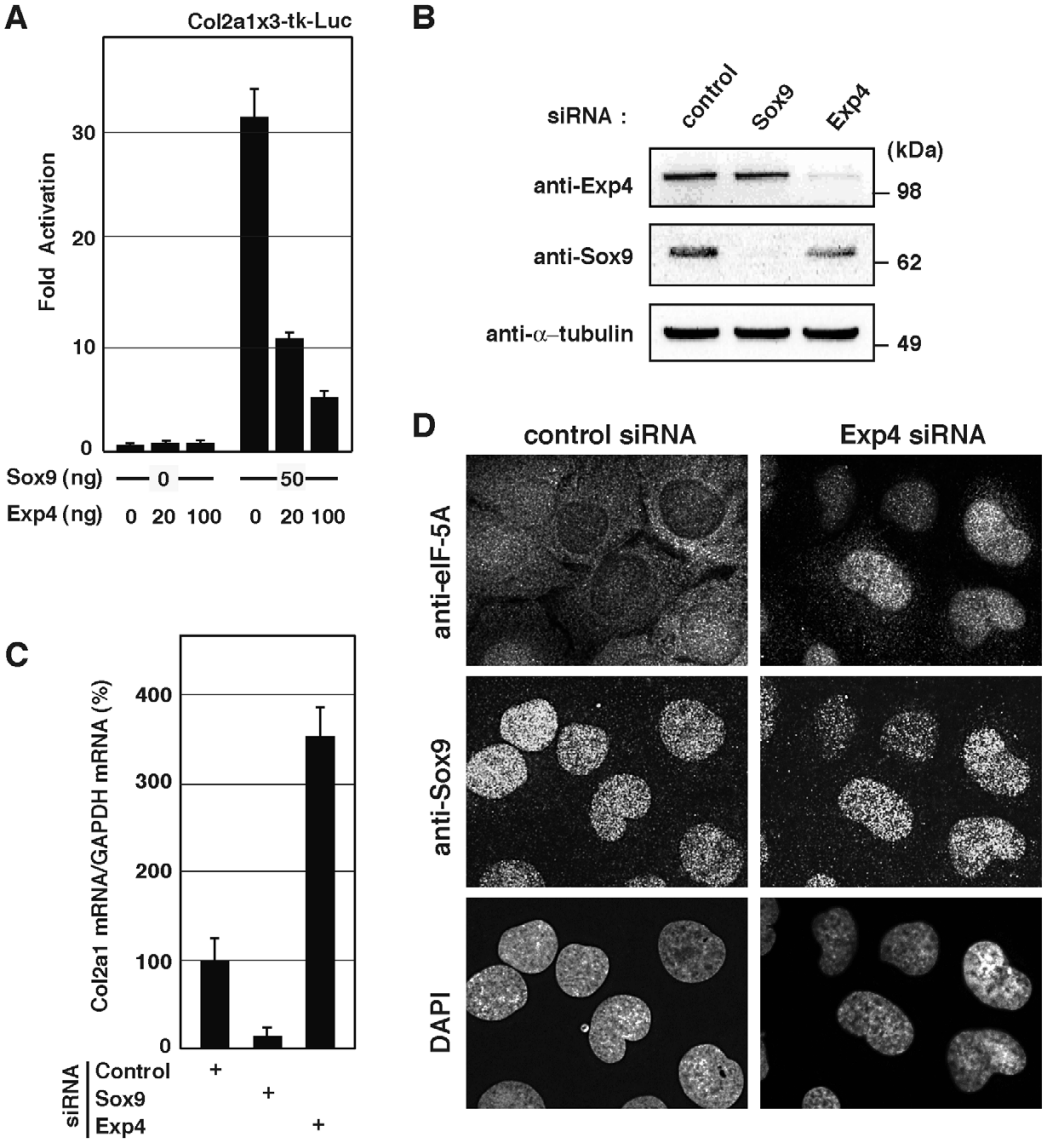
Effects of Exp4 on the localization of Sox9 and Sox9-mediated transcription. (A) The effect of Exp4 on Sox9-mediated transcription. HEK293 cells were transiently transfected with the luciferase reporter gene (Col2a1x3-tk-Luc) and the indicated amounts (ng) of Sox9 and Exp4. Values are the mean ± SD of at least three experiments. (B) The knockdown efficiency of siRNA for Sox9 and Exp4. U2OS cells were treated with control siRNA, siRNA for Sox9, or siRNA for Exp4. The efficacy of siRNA was monitored by detecting protein levels by Western blotting. (C) The effect of Exp4 depletion on Sox9 target gene expression. U2OS cells were treated with the indicated siRNAs and the mRNA for *Col2a1* was examined by qRT-PCR. The relative amount of Col2a1 mRNA is shown: the amount of Col2a1 mRNA from the control siRNA cells is set at 100. Values are the mean ± SD of at least three experiments. (D) The effect of Exp4 depletion on the localization of endogenous Sox9. U2OS cells were treated with siRNA for Exp4 or control siRNA. Cells were incubated with anti-Sox9 antibody and anti-eIF-5A, and visualized using an Alexa488-conjugated secondary antibody and a Cy3-conjugated secondary antibody. Nuclei were visualized by staining with DAPI. The same cells are shown for each of the siRNAs.

To elucidate how Exp4 affects the functions of Sox9, we first examined whether ectopically expressed Exp4 reduced Sox9-mediated activation of transcription. As the reporter gene, we used a DNA construct containing three repeats of the *Col2a1* enhancer sequence that binds Sox9 and the thymidine kinase (tk) promoter fused to the firefly luciferase (Luc) gene (Col2a1x3-tk-Luc). In the presence of Sox9, transcription of the reporter gene dramatically increased (compare bars of Sox9 “0 ng” with those of “50 ng” in Figure 3A). However, the increase in transcriptional activation was significantly reduced in the presence of Exp4, and this reduction was dose-dependent (compare the right three bars of Exp4 0, 20, and 100 ng in Figure 3A). These results suggest that Exp4 effectively suppresses the transcriptional activity of Sox9.

To confirm the suppressive function of Exp4 in physiological conditions, we performed loss-of-function studies with siRNAs in U2OS cells, which endogenously express Exp4 and Sox9: our method of siRNA knockdown for Exp4 or Sox9 successfully depleted these proteins from the cells (Figure 3B). We first examined the effect of Exp4 on the transcriptional activity of Sox9 on the *Col2a1* gene, which is a well-known direct target gene of Sox9. As expected, depletion of Sox9 significantly reduced transcription of this endogenous target gene (Figure 3C). Depletion of Exp4 enhanced transcription of the *Col2a1* gene by 3-fold (Figure 3C). These results indicate that Exp4 suppresses the transcriptional activator function of Sox9. Since Exp4 is known to be involved in both nuclear import [22] and export [20], [21], this raises the possibility that Exp4 affects the transcriptional activity of Sox9 through re-localization of Sox9. Thus, we examined whether siRNA knockdown of Exp4 alters the intracellular localization of Sox9 in U2OS cells. As it has been shown that Exp4 exports eIF-5A from the nucleus [20], we used eIF-5A as an internal control for the depletion of Exp4. Treatment with siRNA for Exp4 clearly induced nuclear localization of eIF-5A (Figure 3D), but had no effect on Sox9 localization (Figure 3D). These results indicate that the effects of Exp4 on the transcriptional activity of Sox9 do not involved changes in Sox9 localization.

### Exp4 inhibits Sox9 transcriptional activity through the HMG box

**Figure 4:**
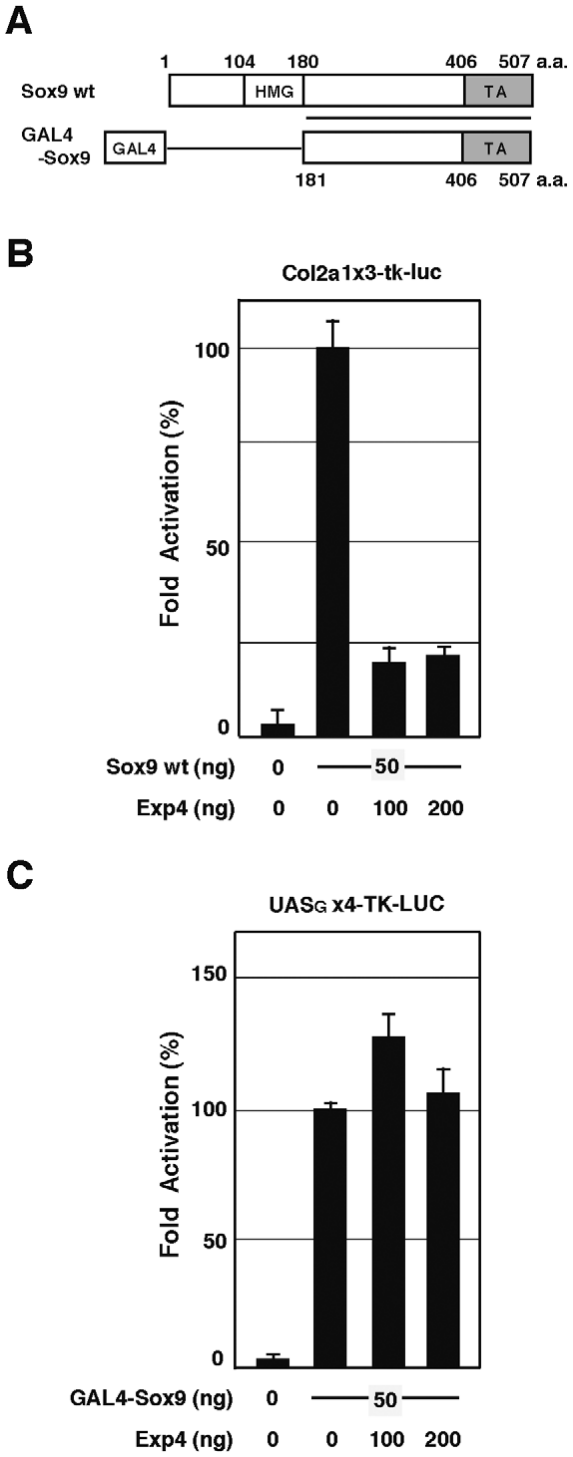
Replacing the HMG box of Sox9 abolished the inhibitory effect of Exp4 on Sox9 transcription. (A) Schematic representation of GAL4-fused Sox proteins (GAL4-Sox9) used in this study. The TA domain is shown as a light gray box (406–507 a.a.). (B) The effect of Exp4 on Sox9-mediated transcription of the Col2a1 reporter gene (Col2a1x3-tk-Luc). HEK293 cells were transiently transfected with the reporter gene and the indicated amounts (ng) of Sox9 or Exp4. The relative fold changes in the luciferase activities are shown: luciferase activity in the presence of 50 ng of Sox9 and the absence of Exp4 is set at 100. Values are the mean ± SD of at least three experiments. (C) The effect of Exp4 on GAL4-Sox9 mediated transcription of the GAL4 reporter gene (UAS_G_x4-TK-LUC). HEK293 cells were transiently transfected with the reporter gene and the indicated amounts (ng) of GAL4-Sox9 or Exp4. The relative fold changes in the luciferase activities are shown: luciferase activity in the presence of 50 ng of GAL4-Sox9 and the absence of Exp4 is set at 100. Values are the mean ± SD of at least three experiments.

To test whether the suppression of Sox9 transcriptional activity depends on the Sox9 HMG box, we made a GAL4-Sox9 fusion construct in which the HMG box of Sox9 (181–507 a.a.) was replaced with the GAL4 DNA-binding domain; the GAL4-Sox9 fusion construct retained the wild-type TA domain (406–507 a.a.) of Sox9 [16] (Figure 4A). As the reporter gene for GAL4-Sox9, we used a DNA construct containing 4 repeats of the GAL4 binding sequence (upstream activating sequence) and the tk promoter fused to the firefly luciferase gene (UAS_G_×4-TK-LUC) [23]. The wild type Sox9 construct was co-transfected into HEK293 cells with Col2a1x3-tk-Luc, or the GAL4-Sox9 construct was co-transfected into HEK293 cells with UAS_G_×4-TK-LUC. In the presence of full-length wild type Sox9, transcription of the Col2a1x3-tk-Luc reporter gene increased and the activation was significantly reduced in the presence of Exp4 (Figure 3A, 4B). In contrast, while the transcription of the UAS_G_×4-TK-LUC reporter gene significantly increased in the presence of GAL4-Sox9, the activation was not reduced in the presence of Exp4 (Figure 4C). These data indicate that the HMG box of Sox9 is essential for Exp4 dependent suppression, and suggest that Exp4 has a direct role in suppression of Sox9 through the HMG box. This binding scheme distinguishes Exp4 from the known co-regulators which bind to the TA domain of Sox9.

### Exp4 inhibits the DNA-binding activity of Sox9

**Figure 5:**
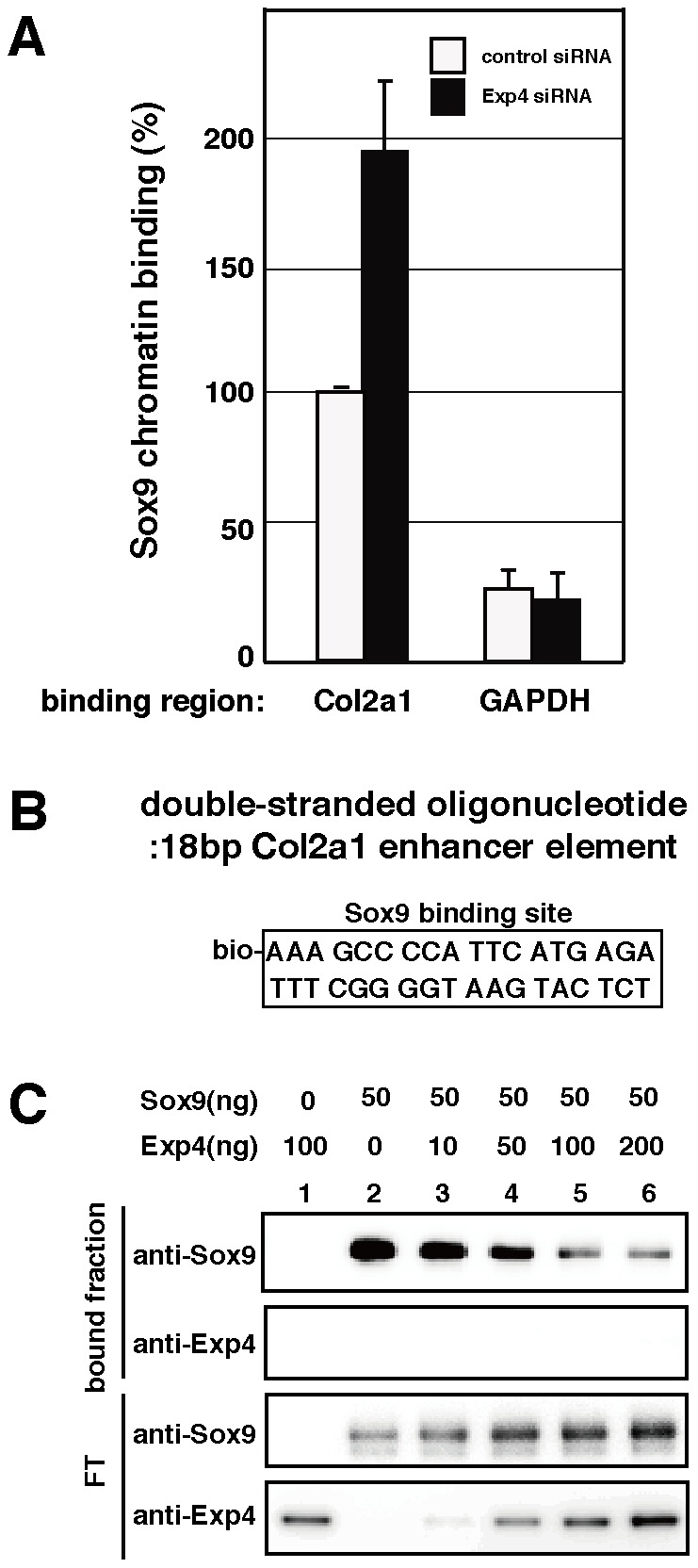
Effect of Exp4 on Sox9 binding to its target gene. (A) The effect of Exp4 depletion on the recruitment of Sox9 to its endogenous target gene. U2OS cells were treated with control siRNA (open bar) or siRNA for Exp4 (solid bar). After a 48 h incubation, fixed chromatin was prepared from both groups of treated cells and subjected to immunoprecipitation with anti-Sox9 antibody. The *Col2a1* enhancer regions recovered in the two precipitated fractions were quantified by PCR. As a control, *GAPDH* genes recovered in the two precipitated fractions were quantified. The relative amount of enhancer DNA is shown: the amount of *Col2a1* enhancer DNA obtained from the control siRNA cells is set at 100. (B) The DNA sequence of the probe used for DNA pull-down contains a biotinylated 18 base-pair Sox9 binding sequence of the *Col2a1* enhancer region. (C) DNA pull-down assay with the indicated amounts (ng) of His-Sox9 and FLAG-Exp4. His-Sox9 and FLAG-Exp4 were incubated with a 5′ biotinylated double-stranded DNA probe. The complex was pulled down with streptavidin-agarose and the streptavidin-agarose bead bound fraction and flow-through fractions (FT) were analyzed by Western blotting.

As Exp4 suppresses Sox9-mediated transcription through its HMG box, this raises the possibility that Exp4 might inhibit DNA binding of Sox9 to its target DNA sequence. To examine this possibility, we treated U2OS cells with siRNA for Exp4 and performed chromatin immunoprecipitation (ChIP) assays with anti-Sox9 antibody or control antibody. The recovered chromatin was amplified using primers specific for *Col2a1*. In Exp4 knockdown cells, recruitment of endogenous Sox9 to the *Col2a1* gene was 1.9-fold higher than in control cells (Figure 5A). These results suggest that Exp4 suppresses the DNA-binding activity of Sox9 *in vivo*.

To further confirm that Exp4 inhibits the DNA binding activity of Sox9, we performed a DNA pull-down assay using purified recombinant Sox9 and Exp4 proteins *in vitro*. A biotinylated 18 bp sequence of the *Col2a1* intronic enhancer element was used as described in *Materials and Methods* (Figure 5B). Sox9 proteins bound to the intronic enhancer sequence (lane 2 in Figure 5C), and Exp4 inhibited the binding of Sox9 to the sequence in a dose-dependent manner (lanes 3–6 in Figure 5C). These results indicate that Exp4 suppresses the transcriptional activity of Sox9 by inhibiting its DNA binding activity.

## Discussion

In the present study, we purified proteins which stably interact with Sox9, and identified Exp4 as a major interacting partner protein of Sox9. Our results show that Exp4 controls Sox9-mediated transcriptional activity by modulating the DNA binding ability, but not the intracellular localization, of Sox9.

### Roles of Exp4 in suppression of Sox9-mediated transcriptional activation

Exp4 is a member of the exportin family. Exportins belong to the importin β superfamily of transport proteins. Exportins are known to export cargo (RNAs and/or proteins) from the nucleus to the cytoplasm by binding cargo in the presence of RanGTP: Exp4 exports eIF-5A and Smad3 to the cytoplasm in the presence of RanGTP [20], [21]. However, Exp4 seems to have different modes of transport. It has been reported that Exp4 can also import Sox2 and SRY proteins into the nucleus in a Ran-independent manner [22]. Interestingly, Görlich and his colleagues have reported that thymidylate synthase preferentially binds Exp4 in the absence of Ran and its binding does not affect localization of the protein [20]. Likewise, our results showed that Exp4 directly bound Sox9 *in vitro* in the absence of RanGTP, and that Exp4 does not affect the cellular localization of Sox9.

The depletion of Exp4 clearly increased the binding of Sox9 to its target gene and increased the expression of the gene without altering the cellular localization of Sox9. These findings raise the possibility that Exp4 has an additional function when it binds to Sox9 in the nucleus. Previous reports suggest that the importin β family may act as chaperone proteins in preventing aggregation of highly charged basic proteins such as ribosomal proteins and histones prior to binding to nucleic acids [24], [25]. This suggests that importin family proteins modulate DNA binding activity by neutralizing highly charged DNA binding domains. Since the HMG box of Sox9 is also a highly charged DNA binding domain containing a number of Lysine and Arginine residues, Exp4 may act as a “neutralization factor” or “chaperone” for the highly charged HMG box of Sox9 and repress the function of Sox9 by suppressing its ability to bind to its target DNA.

There was, however, another possible means by which Exp4 could suppress Sox9 transcription: by exporting cofactors required for Sox9-mediated transcription out of the nucleus. To test this possibility, we replaced the HMG box of Sox9 with the DNA binding domain of GAL4, leaving the C-terminal intact. It is known that the C-terminal of Sox9 bears the transactivating function [16], [17]. Mutations in this domain cause the Sox9-dependent disease “campomelic dysplasia” with skeletal dysmorphology/sex reversal, and this disease is due to loss of transcription activation function [26]. Therefore, if Exp4 removed essential cofactors, such as Smad3, by exporting them out of the nucleus, GAL4-Sox9 would loose transcription activation function in the presence of Exp4. This was not the case (Figure 4C); Exp4 did not affect transcription activity in the absence of the N-terminal HMG domain. This finding supports the idea that Exp4 directly regulates Sox9 function through the HMG box and not through the export of Sox9 cofactors.

### A functional link between DNA-binding and NLS/NES

Previous studies have reported that many types of transcription factors possess an NLS or NES near their DNA binding domains. Examples include helix–loop–helix [27], Zn finger [28], Forkhead [29] and HMG box [2] transcription factors. Interestingly, Cokol et al. found by *in silico* analysis that about 90% of the DNA binding regions overlap a NLS region [30]. This supports our hypothesis that factors which bind a NLS and/or NES can regulate transcriptional activity by modulating binding to DNA.

This hypothesis is supported by three examples. The first example is interaction between a 14-3-3 protein complex, which works as a molecular scaffold, and the Forkhead DNA binding domain of FOXO4. FOXO4 has three 14-3-3 binding sites, and the Forkhead DNA binding domain lies between the 5′ site and the middle site. Moreover, the middle 14-3-3 binding site overlaps the FOXO4 NLS. Binding of the 14-3-3 complex to the 5′ and middle sites of FOXO4, completely inhibits the binding of FOXO4 to its target DNA and causes rapid export of FOXO4 from the nucleus [31]. The second example is the interaction of importins with the yeast transcription factor GAL4. Importin β inhibits GAL4-DNA binding by competing with DNA for binding to GAL4. On the other hand, the GAL4 which does bind to DNA is recognized by importin α, and importin α stabilizes GAL4-DNA binding [28]. The third example is the result presented in this report: Exp4 binds to Sox9 and inhibits Sox9-DNA binding.

Interestingly, our data show that Exp4 prevents Sox9 from binding to the *Col2a1* gene without affecting the localization of Sox9. This prompts us to speculate that transcriptional factors such as Sox9 may have two forms in the nucleus, a chromatin-bound form and an unbound form, and that these two forms of Sox9 exist in an Exp4-mediated equilibrium. Such dynamic exchange of transcriptional factors must be precisely and temporally regulated by co-regulators. Thus, we propose that Exp4 is a transcriptional co-regulator which controls the initiation rate of Sox9-mediated transcription by preventing DNA binding by Sox9.

## Materials and Methods

### Purification of proteins which interact with Sox9

Recombinant FLAG-tagged Sox9 protein was expressed in a baculovirus expression system in Sf21 cells as previously reported [32]. Briefly, the cells were lysed in B400 buffer (20 mM Tris-HCl (pH 8.0), 400 mM KCl, 10% glycerol, 5 mM MgCl_2_, 0.1% Tween20 and complete protease inhibitor cocktail (Roche, Mannheim, Germany)), and the FLAG-tagged Sox9 was collected with anti-FLAG M2 agarose (Sigma, St Louis, MO, USA). The agarose beads were washed with B100 buffer (20 mM Tris-HCl (pH 8.0), 100 mM KCl, 10% glycerol, 5 mM MgCl_2_, 0.1% Tween20 and complete protease inhibitor cocktail), and were incubated with nuclear extract prepared from HeLa S3 cells [33] for 3 hr at 4°C. The beads were then washed five times with B150 buffer (20 mM Tris-HCl (pH 8.0), 150 mM KCl, 10% glycerol, 5 mM MgCl_2_, 0.1% Tween20 and complete protease inhibitor cocktail), and the proteins bound to the beads were eluted with 0.1 M glycine-HCl (pH 2.5) and separated on 4% to 12% polyacrylamide gels (NuPAGE, Invitrogen, Carlsbad, CA, USA). The proteins recovered from the gel were analyzed by mass spectrometry.

### Plasmids

The full-length cDNA for human Exp4 was obtained as follows. The 5′ region of Exp4 in the Celera 5′ EST Clone 19600417490688 (Invitrogen) was removed by digestion with Asp718/EcoRI and then inserted into the corresponding sites in pBluescript SK+ (Stratagene, La Jolla, CA, USA). The 3′ region of Exp4 in IMAGE Clone 1625054 (GenBank accession no. AA993090, Invitrogen) was removed by digestion with EcoRI/NotI and inserted into the corresponding sites of the pBluescript SK+ plasmid containing the 5′ region of Exp4. Finally, the middle region of Exp4 was removed from IMAGE Clone 614088 (GenBank accession no. BC050680, Invitrogen) by digestion with EcoRI and inserted into the relevant sites of the pBluescript SK+ plasmid containing the 5′ and 3′ regions of Exp4. The resulting full-length Exp4 cDNA was subcloned into pFASTBAC for protein production in a baculovirus system and into pCAGGS [34] for expression in mammalian cells. Mouse Sox2 (Sox2) and mouse Sox11 (Sox11) were constructed with cDNAs that had been generously provided by Dr. Hisato Kondoh (Osaka University, Osaka, Japan), and N-terminal HA-tags fused in-frame with Sox2, Sox11, and Sox9 [35] were inserted into the pcDNA3 vector (Invitrogen). The luciferase reporter plasmid, Col2a1x3-tk-Luc, was constructed so as to contain three copies of the *Col2a1* enhancer element directly upstream of the tk basal promoter. pCMX-GAL4 [36] encodes the GAL4 DNA binding domain (1–147 a.a.). pCMX-GAL4-Sox9 was constructed by inserting a PCR-amplified DNA fragment encoding amino acids 181 to 507 of mouse Sox9 into pCMX-GAL4 cleaved with Asp718 and BamHI. The luciferase reporter plasmid, UAS_G_×4-TK-LUC [23], was used with GAL4-Sox9. The sequences of all of the plasmids used in this study were checked using an ABI prism310 DNA sequencer (Applied Biosystems, CA, USA).

### Preparation of Anti-Exp4 Antibody

His-tagged Exp4 (His-Exp4, 931–1125 a.a.) corresponding to the C-terminal region of human Exp4 was cloned into pET28a (Stratagene) and expressed in *Escherichia coli* BL21 (DE3). The *E. coli* were lysed in G buffer containing 6 M guanidine-HCl, 20 mM Tris-HCl (pH 8.0), 500 mM KCl, 10% glycerol, 5 mM MgCl_2_, 0.1% Tween 20, and 20 mM Imidazol. The lysates were mixed with Ni^2+^ resin (Sigma) at room temperature for 1 hr. Then, His-Exp4 was eluted with G buffer containing 200 mM Imidazole. The protein solution was subjected to dialysis with buffer containing 50 mM Tris-HCl (pH 8.0), and 150 mM NaCl to remove guanidine. Polyclonal Exp4 antibodies were produced by Sigma-Aldrich Life Science Japan using His-Exp4 protein as an antigen, produced as described above. Anti-Exp4 antibody was purified by affinity to an antigen-conjugated column and was used for immunoblotting to test its specificity.

### Cell culture, transient transfection, and reporter assay

HeLa S3 cells (ATCC CCL-2.2; American Type Culture Collection), HEK293 cells (ATCC-CRL-1573) and U2OS cells (ATCC HTB-96) were grown in Dulbecco's modified Eagle's medium (DMEM) (Sigma) supplemented with 10% fetal bovine serum (JRH Sciences, Lenexa, KS) and 1×penicillin-streptomycin-glutamine (Invitrogen) at 5% CO_2_ and 37°C. For reporter assays, 5×10^4^ HEK293 cells were seeded into 24-well plates and incubated for 24 hr before transfection with Lipofectamine 2000 reagent (Invitrogen) according to the manufacturer's protocol. Cells were co-transfected with three plasmids: the Sox9/GAL4-Sox9 expression plasmid, the Exp4 expression plasmid, and 200 ng of luciferase reporter plasmid. The total amounts of the transfected plasmids were adjusted to 600 ng with an empty vector plasmid. 50 ng of pRL-SV40 (Promega, Madison, WI, USA) was used as an internal control to normalize the transfection efficiencies. The cells were harvested 36 hr after transfection and the cell lysates were subjected to luciferase assay using the Dual-Luciferase Assay System (Promega). All transfection experiments were performed in triplicate.

### Immunoprecipitation and Western blotting

To detect the interaction of endogenous Sox9 and Exp4 in cells, nuclear extracts from U2OS cells were incubated with anti-Sox9 antibody kindly provided by Prof. K. Morohashi (Kyushu University, Fukuoka, Japan) at 4°C for 1 hr. Normal rabbit IgG was used as a control. Immune complexes with anti-Sox9 antibodies were recovered with protein A-Sepharose (GE Healthcare UK Ltd., UK) and subjected to Western blotting analysis using anti-Exp4 antibody. To detect the interaction of Exp4 and Sox family members in cultured cells, the HA-tagged expression plasmids for the Sox family members were co-transfected into HEK293 cells for 36 hr with the Exp4 expression plasmid. The cells were lysed and incubated with anti-HA (12CA5) antibody (Roche) conjugated protein A-Sepharose. The immunoprecipitates were separated on NuPAGE and subjected to Western blotting analysis using anti-Exp4 antibody and anti-HA (12CA5) antibody.

### GST pull-down assays

Various regions of Sox9 were amplified by PCR, inserted into pGEX-6P-1 (GE Healthcare UK Ltd.), and expressed as GST-fused proteins in *E.coli.* The *E.coli* were sonicated in B400 buffer. After centrifugation at 70,000×g for 20 min, the supernatants were incubated at 4°C for 3 hr with glutathione-Sepharose (GE Healthcare UK Ltd.). After the incubation, the glutathione-Sepharose was washed three times with B400 buffer, washed two times with B100 buffer, and then incubated with nuclear extracts from HeLa cells at 4°C for 2 hr. The proteins bound to the Sepharose were eluted by lithium dodecyl sulfate (LDS) sample buffer (Invitrogen) and subjected to Western blotting analysis using anti-Exp4 antibody. The GST-fused proteins including the HMG box regions of Sox2, Sox9 and Sox11 were expressed in *E.coli* and prepared as described above. Each GST-fused protein solution was incubated with purified recombinant FLAG-Exp4 protein and glutathione Sepharose at 4°C for 2 hr. The proteins bound to the Sepharose were eluted by LDS sample buffer and subjected to Western blotting analysis with anti-Exp4 antibody.

### Small interfering RNA treatments and Real-time PCR (qRT-PCR)

2×10^5^ U2OS cells were seeded into 6-well plates. After 24 hr, the cells were transfected with 50 nM siRNA duplexes for Exp4 or for Sox9 (Sigma) using 5 µl Lipofectamine RNAiMax reagent (Invitrogen). A control siRNA (Mission siRNA Universal Negative Control, Sigma) was used as a negative control. After a 6 hr incubation with the transfection reagents, the cells were cultured in fresh culture medium for 30 hr. Total RNA was prepared from the cells using a Fastpure RNA kit (Takara bio Inc., Japan), and was reverse-transcribed using a high-capacity RNA-to-cDNA Master Mix (Applied Biosystems). qRT-PCR was performed with an ABI PRISM 7700 Sequence Detection System (Applied Biosystems). *Power* SYBR Green (Applied Biosystems) was used as a fluorescent dye. Specific primers for the *Col2a1* and *GAPDH* genes were as follows (5′ to 3′): *Col2a1*, forward: CCGGGCAGAGGGCAATAGCAGGTT; reverse: CATTGATGGGGAGGCGTGAG; *GAPDH*, forward: CGGAGTCAACGGATTTGGTCGTAT; reverse: AGCCTTCTCCATGGTGGTGAAGAC.

### Immunohistochemistry of siRNA transfected cells

For microscopic observation of siRNA transfected cells, 1×10^5^ U2OS cells were seeded into 35 mm glass bottom culture plates, and 12 hr later U2OS cells were transfected with 50 nM siRNA for Exp4 (Sigma) using Lipofectamine RNAiMax reagent according to the manufacturer's protocol. The control siRNA (Mission siRNA Universal Negative Control; Sigma) was used as a control. After a 6 hr incubation with the transfection reagents, the cells were cultured in fresh medium for 42 hr. The cells were fixed with 4% formaldehyde in phosphate-buffered saline (PBS) for 30 min at room temperature and washed three times with PBS containing 0.1% TritonX-100 (PBSTX). The cells were then incubated with Blocking One (Nakarai tesque Inc., Japan) for 30 min. The cells were incubated with anti-eIF-5A antibody (Cell Applications Inc., CA, USA, 1∶200 dilution) and anti-Sox9 antibody (Invitrogen, 1∶500 dilution) for 16 hr at 4°C, washed three times with PBSTX, and then, incubated with Alexa488-conjugated anti-rabbit IgG (Invitrogen, 1∶2000 dilution) and Cy3-conjugated anti-mouse IgG (GE, 1∶2000 dilution) at room temperature for 2 hr. Finally, the cells were washed three times with PBSTX, incubated with 50 ng/ml 4′,6-diamidino-2-phenylindole (DAPI) for 10 min, and mounted with 60% glycerol-PBS for fluorescence microscopy. Cells were analyzed and photographed with a DeltaVision RT system (Applied Precision Inc., WA, USA).

### ChIP Assays

After cross-linking U2OS cells with 0.75% formaldehyde for 10 min at room temperature, cells were rinsed twice with cold PBS, harvested in lysis buffer containing 50 mM Tris-HCl (pH 8.0), 10 mM EDTA and 1% SDS, and incubated for 5 min at room temperature. Their chromatin was sonicated to generate DNA fragments of 500–1000 base pairs. The chromatin solution was diluted by 0.1% SDS with dilution buffer containing 20 mM Tris-HCl (pH 8.0), 2 mM EDTA, 150 mM NaCl and 1% TritonX-100, and incubated with normal rabbit IgG or anti-Sox9 antibody, and Dynabeads Protein A (Invitrogen) overnight at 4°C. Next, Dynabeads Protein A adsorbed to the immunocomplexes were collected and washed three times with 50 mM Tris-HCl (pH 8.0), 5 mM EDTA, 150 mM NaCl, 0.5% Nonidet P-40, 0.1% SDS and Protease inhibitor Cocktail, and finally the chromatin fragments were eluted from the beads with 1% SDS in 0.1 M NaHCO_3_. After the cross-linking was reversed by heating overnight at 65°C, DNA fragments were recovered using a QIAquick Gel Extraction Kit (Qiagen, Japan). The purified DNA fragments were used for qRT-PCR with an ABI PRISM 7700 Sequence Detection System. *Power* SYBR Green was used as a fluorescent dye. Specific primers for the *Col2a1* and *GAPDH* genes were as follows (5′ to 3′): *Col2a1*, forward: ACCTGTGAATCGGGCTCTGT; reverse: CCCACTGGACCTCGTCTCTC; *GAPDH*, forward: GGTTTACATGTTCCAATATGATTCCA; reverse: AAGATGGTGATGGGATTTCCAT.

### Protein-DNA binding analysis

His-tagged Sox9 protein was expressed in a baculovirus expression system as described previously [35]. The cells were lysed in B400 buffer. The His-tagged proteins were recovered with a Ni^2+^ Sepharose column using a liquid chromatography system, the ÄKTA explorer10S (GE Healthcare UK Ltd). An equal quantity of biotinylated sense (5′-bio-aaagccccattcatgaga-3′) and un-biotinylated anti-sense (5′-tctcatgaatggggcttt-3′) DNA were mixed and incubated at 95°C for 5 min. This was then cooled down to room temperature to generate double stranded DNA. 50 µl of streptavidin-agarose (Upstate Biotechnology Inc., NY, USA) was mixed with 100 pmol of double stranded DNA, incubated at 4°C for 30 min, and then, washed with B100 buffer. Next, the agarose beads were incubated with His-Sox9 (0 or 50 ng) and FLAG-Exp4 (0–200 ng) in B100 buffer at 4°C for 3 hr, and washed three times with B100 buffer. Finally, the proteins bound to the DNA were eluted in LDS sample buffer and analyzed by Western blotting with an anti-Exp4 or anti-Sox9 antibody.
